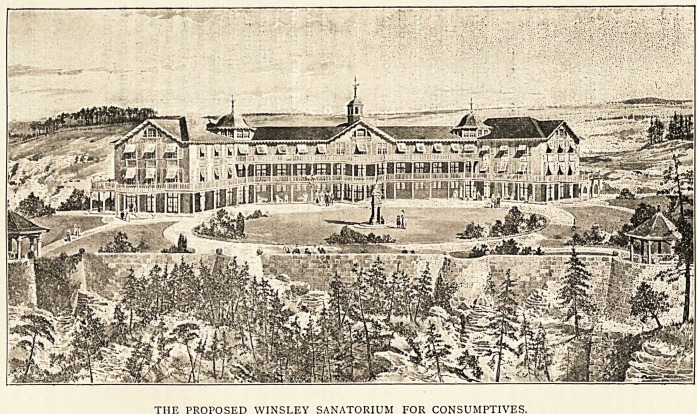# Editorial Notes

**Published:** 1901-06

**Authors:** 


### Editorial Notes.

**The Queen Victoria Jubilee Convalescent Home.**—The first report of this institution, from which extracts were made in our last number (p. 83), has since been widely circulated amongst the subscribers to the fund. It was not possible to send it to all, inasmuch as there were a large number of contributors whose names are not known; but to all those who contributed the sum of £50 and upwards a copy of the report was forwarded. On perusal of the list of the officers and governors, it will be seen that the president (Sir Edward Payson Wills, K.C.B.) with the vice-presidents (Joseph Storrs Fry and Edward Robinson), and the honorary treasurer (Philip Henry Vaughan), may well feel that they have a very representative and experienced staff of governors, who are not likely to be lacking in judgment or care in their management of the institution. The treasurer is most anxious that the full number of beds which the Home will accommodate may be in due time filled, in order that the hospitals may be able to have the full complement of sixty-five beds which was at first proposed, and further that the remaining fifteen beds may be free of charge to suitable patients who have not been admitted from the institutions. It was arranged that for the first year the number of free beds in use by the Bristol Royal Infirmary and the Bristol General Hospital should not exceed twenty-one to each, and the wisdom of this course was shown by the fact that the year closed with a very small balance in hand, the expenditure having been a little over £2,000. The investments are now valued at £70,731, and the income will approximate a sum exceeding £2,500. The treasurer's proposal to increase the endowment fund by a further sum of £30,000 is now before the Bristol citizens, and the governors of the Home are gratified at the adoption by the Lord Mayor of this scheme at the meeting held in the Guildhall on April 26th, 1901, when the following resolution was adopted :—

"That the Lord Mayor be requested to issue an appeal for donations to the Queen Victoria National Memorial, and to a local fund towards completing the endowment of the Queen Victoria Jubilee Convalescent Home."

It was afterwards resolved :—"That the amount subscribed shall be divided between the two funds as follows: one-third of the whole shall be sent to the Lord Mayor of London's fund, and two-thirds be given to the endowment fund of the Convalescent Home, unless specially allocated by the donors."

A General Committee has been appointed to carry these resolutions into effect, and an appeal has been circulated among the citizens, of which the following quotation forms a part:—

"The citizens of Bristol have already expressed their loyal reverence for her late Majesty in the memorial raised for the Diamond Jubilee in the establishment of the Convalescent Home. But a national memorial is so obviously fitting, that I venture to ask that the city of Bristol takes its full share in securing one worthy of our late illustrious monarch. I also feel confident that my fellow-citizens will accord the same hearty acceptance which the town's meeting gave to the proposition to complete the endowment of the Convalescent Home. The amount necessary for these two purposes should not be less than £15,000, and I hope to have the pleasure of publishing the first list of donations on May 24th, Queen Victoria's birthday. An early reply on annexed form will much oblige."

**Winsley Sanatorium for Consumptives.**—Several meetings have been held in the Wiltshire district of the three counties. That at Bath on March 6th, under the presidency of the Mayor (Mr. T. B. Silcock), was addressed by Sir James Crichton Browne, Dr. E. J. Cave, Dr. W. H. Symons, Dr. Lionel Weatherly, and the Rev. Canon Quirk; at Bradford-on-Avon, on March 11th, Dr. W. H. Symons was the principal speaker; at Devizes, on the 27th, Dr. Watson Williams and Dr. Weatherly were the chief speakers; at Trowbridge, on April 3rd, an address was given by Dr. Shingleton Smith; and later still, at Calne, Drs. Weatherly and Michell Clarke spoke on the subject. These meetings have been well attended, and much information has been disseminated. A large meeting was also held recently at Weston-super-Mare, at which Dr. Robert Maguire gave an interesting address. Some lantern slides illustrating the site of the sanatorium were shown by Dr. Weatherly. We congratulate the Hon. Sec. for Weston, Dr. Ballance, on the large and representative gathering present.

We are indebted to Dr. Weatherly for the following description of the site and the proposed building:—"By all who are conversant with the needs as to site and situation of a sanatorium for consumptives, the land chosen by the committee at Winsley has been thought to be *ideal*, and then added to this we have the fact of its being in the centre of the three counties, and with capital railway communication for all parts of these counties. The committee consider themselves very fortunate in having been able to secure so admirable a position upon which to erect their sanatorium. The parish of Winsley contains the hamlets of Murhill, Conkwell and Turley. Conkwell, which stands on the border of Wilts and Somerset, is a most interesting little place. Like Petra of old, it stands in a cleft of the rocks, and is said to exhibit greater marks of antiquity than almost any other cluster

THE PROPOSED WINSLEY SANATORIUM FOR CONSUMPTIVES.

of houses in the kingdom. Its sylvan scenery is very lovely. Murhill Quarry, in which the sanatorium will be built, has a most beautiful outlook over the Wiltshire Downs, with the White Horse at Westbury very distinctly visible. The pretty river Avon, the Avon and Kennet Canal, and the railway, help to keep the scene full of life, and, to my thinking, greatly enhance the value of the situation for the purposes required. The parish of Winsley has for generations been noted for its salubrity. The sanatorium, the plans of which are being prepared by Messrs. Silcock and Reay, architects, of Bath, will be much on the lines of that splendidly-arranged institution, Hohenhonef, which, as many know, is situated on the Siebengebirge, overlooking Roland's Ecke on the Rhine. It will be seen that the building has two wings diverging at a somewhat open angle, and this will afford great protection from the east and west; while the high banks of the Quarry, some of which are already well wooded, will protect it still further, and the rising ground behind shelters it from the north. The woods on the estate are very pretty, and already lovely walks are practically made, and with much tree-planting on the land, there can be no doubt the surroundings of this sanatorium will be hard to beat in this or any other country." A more detailed description of the plans of the building are given on page 187, under the head of "Local Notes."

**The British Congress on Tuberculosis,** which is to be held in London, will sit from July 22nd to the 26th. It is another evidence of the earnest desire to devise more effectual means of coping with tuberculosis, and to at least place it in the same category as cholera, typhus fever, and small-pox, which, though not abolished, are no longer national scourges. Much useful work will be done in the various sections, which will deal with such questions as notification, control of milk and meat supplies, and the provision of sanatoria, climatology, pathology, bacteriology, and statistics. But not the least interesting features of the Congress will be the addresses by Koch of Berlin, Brouardel of Paris, and McFadyean of our own Royal Veterinary College. The subscription for membership of the Congress is £1, and any of our readers wishing to attend should forward that amount to the Secretary-General, 20 Hanover Square, W.

**Organo-therapy.**—It is high time that the revival of the long-discarded practices of the past in relation to the medicinal uses of animal products should receive a vigorous protest. The editor of the *Practitioner* (April, 1901) has done well in devoting the number to a review of what has been and is being done in organo-therapy. No one can deny that the therapeutic uses of thyroid extract are such as to justify the statement of Dr. George R. Murray that its introduction into rational medicine is one of the most solid triumphs of modern science; but the enthusiast has expected great things from it in many other conditions than myxœdema, and in most of these he has been grievously disappointed. If thyroid extract has been for the most part a failure, how much more so are the numerous preparations of animal substances which every manufacturer now provides for the doctor and his patients? Almost every organ and tissue is converted into powder, tabloid, palatinoid, pill, or extract, and each preparation is credited with certain and often miraculous virtues. Well may we wonder whether the druggist and those who use his wares have not all joined the faith-cure brigade. The medico-literary causerie on organo-therapy in antiquity naturally raises the question whether the (*Ibid.*, p. 420) *Natural History* of Pliny the Elder is to be one of the text-books of the twentieth century. The ancients left nothing untried in organ and excrement therapy. We are fast following the example set by them in this matter. Mr. Malcolm Morris remarks that we are making steady progress in the spirit of the apostolic precept, "Try all things."

## Figures and Tables

**Figure f1:**